# Supplementary material for: The Drosophila microbiome has a limited influence on sleep, activity, and courtship behaviors
Source: Sci Rep. 2018 Jul 13;8:10646. doi: 10.1038/s41598-018-28764-5 (PMC6045657; doi:10.1038/s41598-018-28764-5)
Supplement: Supplementary file 1 — Supplementary Figure and Tables [file 41598_2018_28764_MOESM1_ESM.pdf]

The *Drosophila* microbiome has a limited influence on sleep, activity, and courtship behaviors

Joel Selkrig [1, 2, *], Farhan Mohammad [3, 4, *], Soon Hwee Ng [5, 6], Chua Jia Yi [3, 4], Tayfun Tumkaya [4, 7], Joses Ho[4], Yin Ning Chiang [5], Dirk Rieger [8], Sven Pettersson [1, 8], Charlotte Helfrich-Förster [9], Joanne Y. Yew [5, 10, +], Adam Claridge-Chang [3 ,4, 7, +]

1. School of Biological Sciences; Lee Kong Chian School of Medicine, Nanyang Technological University, Singapore.
2. European Molecular Biology Laboratory (EMBL), Genome Biology Unit, Meyerhofstrasse 1, 69117 Heidelberg, Germany.
3. Program in Neuroscience and Behavioral Disorders, Duke-NUS Medical School, Singapore.
4. Institute for Molecular and Cell Biology, Agency for Science Technology and Research, Singapore 138673.
5. Temasek Life Sciences Laboratory; Department of Biological Science, National University of Singapore
6. Centre for Evolutionary Biology, School of Animal Biology, University of Western Australia, Crawley, WA 6009, Australia.
7. Department of Physiology, National University of Singapore, Singapore.
8. Department of Microbiology, Cell and Tumor Biology, Karolinska Institutet, Solna, Sweden
9. Neurobiology and Genetics, Theodor-Boveri Institute, Biocenter, University of Würzburg, 97074 Würzburg, Germany.
10. Pacific Biosciences Research Center, 1993 East West Road, University of Hawaiʻ i at Mānoa, Honolulu, HI 96722, USA.
* These authors contributed equally to this work.
+ Correspondence: claridge-chang.adam@duke-nus.edu.sg and jyew@hawaii.edu

Supplementary figure and tables

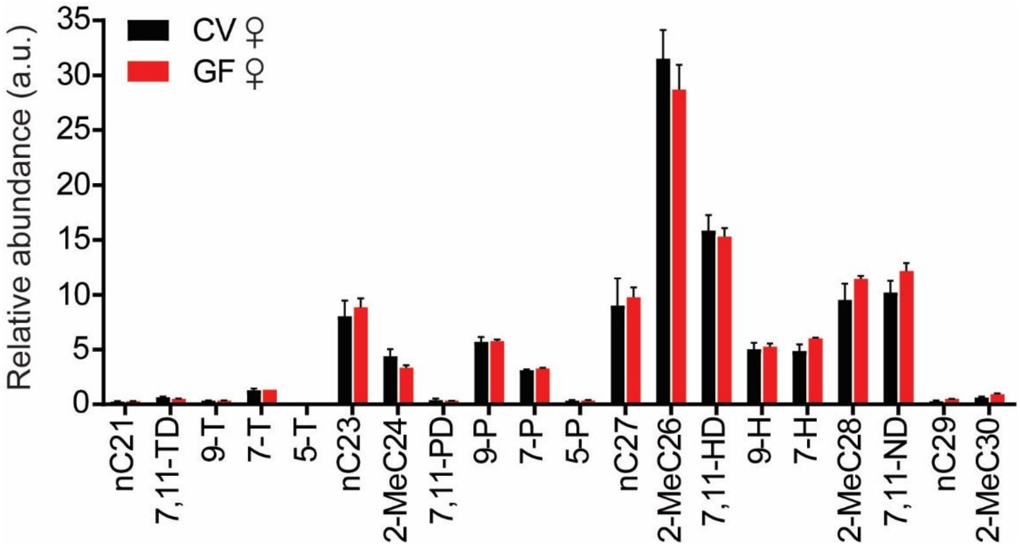

Figure S1. Relative abundance of individual CHC species as measured by GC/MS. Data represent the average signal intensity +/– standard deviation of 3 replicate sets per condition, with each set consisting of 8 females. The area of each CHC peak is normalized to the area of the internal heptacosane standard. Double bond position for alkenes and dienes and branching position for branched compounds are indicated; TD: tricosadiene; PD: pentacosadiene: HD: heptacosadiene; ND: nonacosadiene; T: tricosene; P: pentacosene; H: heptacosene; Me: methyl branched; a.u.: arbitrary units.

Table S1. Summary of bacterial isolates recovered from Canton S *Drosophila*.
PCR amplification of the 16s rRNA genomic locus from individual bacterial colonies isolated from Canton S fly guts after growth on MRS agar. Sequences were generated using the 8FE-F primer.

Top BLASTn hit (Accession)	16s rRNA Sequence
Uncultured *Lactobacillus* sp. gene for 16S ribosomal RNA, partial sequence, clone: wb-13 (LC036256.1)	AGTCGAACGAGCTGCGCCTAATGATAGTTGATGCTTGCATTAACTTGACTTAAGTTAGCA<br>GCGAGTGGCGAACTGGTGAGTAACACGTGGATAACCTGCCCAGAAGAAGGGGATAACACCTGGAAACAGATGCTAATACC<br>GTATAACAACGAAAACCACATGGTTTTCGTTTGAAAGATGGCCTTTGTGCTATCGCTTTTGGATGGATCCGCGGCGCATT<br>AGCTAGTTGGTGAGATAAAGGCTCACCAAGGCAATGATGCGTAGCCGACCTGAGAGGGTAATCGGCCACATTGGGACTGA<br>GACACGGCCCAGACTCCTACGGGAGGCAGCAGTAGGGAATCTTCCACAATGGACGAAAGTCTGATGGAGCAATGCCGCGT<br>GAGTGAAGAAGGGTTTCGGCTCGTAAAACTCTGTTGTTAGAGAAGAACGGGCGTGAGAGTAACTGCTCACGTCGCGACGG<br>TATCTAACCAGAAAGTCACGGCTAACTACGTGCCAGCAGCCGCGGTAATACGTAGGTGGCAAGCGTTGTCCGGATTTATT<br>GGGCGTAAAGCGAGCGCAGGCGGTCTTCTAAGTCTGATGTGAAAGCCTTCGGCTTAACCGGAGAAGTGCATCGGAAACTG<br>GGAAACTTGAGTGCAGAAGAGGACAGTGGAACTCCATGTGTAGCGGTGAAATGCGTAGATATATGGAGGAACACCAGTGG<br>CGAAGGCGGCTGTCTAGTCTGTAACTGACGCTGANGCTCGAAAGCATGGGTAGCAAACAGGATTAGATACCCTGGTAGTC<br>CATGCCGTAAACGATGAGTGCTAGGTGTTGGAGGGTTTCCGCCCTTCAGTGCCGCAGCTAACGCATTAAGCACTCCGCCT<br>GGGGAGTACNACCGCAAGGTTGAAACTCAAAGGAATTGACGGGGACCCGCACAAGCG
Acetobacter pomorum strain BDGP5 chromosome, complete genome (CP023657.1)	AGTCGCACGAAGGTTTCGGCCTTAGTGGCGGACGGGTGAGTAACGCGTAGGTATCT<br>ATCCATGGGTGGGGGATAACACTGGGAAACTGGTGCTAATACCGCATGACACCTGAGGGTCAAAGGCGTAAGTCGCCTGT<br>GGAGGAGCCTGCGTTTGATTAGCTAGTTGGTGGGGTAAAGGCCTACCAAGGCGATGATCAATAGCTGGTTTGAGAGGATG<br>ATCAGCCACACTGGGACTGAGACACGGCCCAGACTCCTACGGGAGGCAGCAGTGGGGAATATTGGACAATGGGGGCAACC<br>CTGATCCAGCAATGCCGCGTGTGTGAAGAAGGTCTTCGGATTGTAAAGCACTTTCGACGGGGACGATGATGACGGTACCC<br>GTAGAAGAAGCCCCGGCTAACTTCGTGCCAGCAGCCGCGGTAATACGAAGGGGGCTAGCGTTGCTCGGAATGACTGGGCG<br>TAAAGGGCGTGTAGGCGGTTTGTACAGTCAGATGTGAAATCCCCGGGCTTAACCTGGGAGCTGCATTTGATACGTGCAGA<br>CTAGAGTGTGAGAGAGGGTTGTGGAATTCCCAGTGTAGAGGTGAAATTCGTAGATATTGGGAAGAACACCGGTGGCGAAG<br>GCGGCAACCTGGCTCATTACTGACGCTGAGGCGCGAAAGCGTGGGGAGCAAACAGGATTAGATACCCTGGTAGTCCACGC<br>TGTAAACGATGTGTGCTAGATGTTGGGTGACTTAGTCATTCAGTGTCGCAGTTAACGCGTTAAGCACACCGCCTGGGGGA<br>GTACGGCCGCAAGGTTGAAACTCAAAGGAATTGACGGGGGCCCGCACAAGCG
Acetobacter pomorum strain BDGP5 chromosome, complete genome (CP023657.1)	AGTCGCACGAAGGTTTCGGCCTTAGTGGCGGACGGGTGAGTAACGCGTAGGTATCTATC<br>CATGGGTGGGGGATAACACTGGGAAACTGGTGCTAATACCGCATGACACCTGAGGGTCAAAGGCGTAAGTCGCCTGTGGA<br>GGAGCCTGCGTTTGATTAGCTAGTTGGTGGGGTAAAGGCCTACCAAGGCGATGATCAATAGCTGGTTTGAGAGGATGATC<br>AGCCACACTGGGACTGAGACACGGCCCAGACTCCTACGGGAGGCAGCAGTGGGGAATATTGGACAATGGGGGCAACCCTG<br>ATCCAGCAATGCCGCGTGTGTGAAGAAGGTCTTCGGATTGTAAAGCACTTTCGACGGGGACGATGATGACGGTACCCGTA<br>GAAGAAGCCCCGGCTAACTTCGTGCCAGCAGCCGCGGTAATACGAAGGGGGCTAGCGTTGCTCGGAATGACTGGGCGTAA<br>AGGGCGTGTAGGCGGTTTGTACAGTCAGATGTGAAATCCCCGGGCTTAACCTGGGAGCTGCATTTGATACGTGCAGACTA<br>GAGTGTGAGAGAGGGTTGTGGAATTCCCAGTGTAGAGGTGAAATTCGTAGATATTGGGAAGAACACCGGTGGCGAAGGCG<br>GCAACCTGGCTCATTACTGACGCTGAGGCGCGAAAGCGTGGGGAGCAAACAGGATTAGATACCCTGGTAGTCCACGCTGT<br>AAACGATGTGTGCTAGATGTTGGGTGACTTAGTCATTCAGTGTCGCAGTTAACGCGTTAAGCACACCGCCTGGGGAGTAC<br>GGCCGCAAGGTTGAAACTCAAAGGAATTGACGGGGGCCCGCACAAGCG
Acetobacter pomorum strain BDGP5 chromosome, complete genome (CP023657.1)	AGTCGCACGAAGGTTTCGGCCTTAGTGGCGGACGGGTGAGTAACGCGTANNNNNNNNN<br>CCNTGGGTGGGGGATAACACTGGGAAACTGGTGCTAATACCGCATGACACCTGAGGGTCAAAGGCGTAAGTCGCCTGTGG<br>AGGAGCCTGCGTTTGATTAGCTAGTTGGTGGGGTAAAGGCCTACCAAGGCGATGATCAATAGCTGGTTTGAGAGGATGAT<br>CAGCCACACTGGGACTGAGACACGGCCCAGACTCCTACGGGAGGCAGCAGTGGGGAATATTGGACAATGGGGGCAACCCT<br>GATCCAGCAATGCCGCGTGTGTGAAGAAGGTCTTCGGATTGTAAAGCACTTTCGACGGGGACGATGATGACGGTACCCGT<br>AGAAGAAGCCCCGGCTAACTTCGTGCCAGCAGCCGCGGTAATACGAAGGGGGCTAGCGTTGCTCGGAATGACTGGGCGTA<br>AAGGGCGTGTAGGCGGTTTGTACAGTCAGATGTGAAATCCCCGGGCTTAACCTGGGAGCTGCATTTGATACGTGCAGACT<br>AGAGTGTGAGAGAGGGTTGTGGAATTCCCAGTGTAGAGGTGAAATTCGTAGATATTGGGAAGAACACCGGTGGCGAAGGC<br>GGCAACCTGGCTCATTACTGACGCTGAGGCGCGAAAGCGTGGGGAGCAAACAGGATTAGATACCCTGGTAGTCCACGCTG<br>TAAACGATGTGTGCTAGATGTTGGGTGACTTAGTCATTCAGTGTCGCAGTTAACGCGTTAAGCACACCGCCTGGGGAGTA<br>CGGCCGCAAGGTTGAAACTCAAAGGAATTGACGGGGGCCCGCACAAGCG
Uncultured *Lactobacillus* sp. gene for 16S ribosomal RNA, partial sequence, clone: wb-13 (LC036256.1)	AGTCGAACGAGCTGCGCCTAATGATAGTTGATGCTTGCATTAACTTGACTTAAGTTAGCA<br>GCGAGTGGCGAACTGGTGAGTAACACGTGGATAACCTGCCCAGAAGAAGGGGATAACACCTGGAAACAGATGCTAATACC<br>GTATAACAACGAAAACCACATGGTTTTCGTTTGAAAGATGGCCTTTGTGCTATCGCTTTTGGATGGATCCGCGGCGCATT<br>AGCTAGTTGGTGAGATAAAGGCTCACCAAGGCAATGATGCGTAGCCGACCTGAGAGGGTAATCGGCCACATTGGGACTGA<br>GACACGGCCCAGACTCCTACGGGAGGCAGCAGTAGGGAATCTTCCACAATGGACGAAAGTCTGATGGAGCAATGCCGCGT<br>GAGTGAAGAAGGGTTTCGGCTCGTAAAACTCTGTTGTTAGAGAAGAACGGGCGTGAGAGTAACTGCTCACGTCGCGACGG<br>TATCTAACCAGAAAGTCACGGCTAACTACGTGCCAGCAGCCGCGGTAATACGTAGGTGGCAAGCGTTGTCCGGATTTATT<br>GGGCGTAAAGCGAGCGCAGGCGGTCTTCTAAGTCTGATGTGAAAGCCTTCGGCTTAACCGGAGAAGTGCATCGGAAACTG<br>GGAAACTTGAGTGCAGAAGAGGACAGTGGAACTCCATGTGTAGCGGTGAAATGCGTAGATATATGGAGGAACACCAGTGG<br>CGAAGGCGGCTGTCTAGTCTGTAACTGACGCTGANGCTCGAAAGCATGGGTAGCAAACAGGANTAGATACCCTGGTAGTC<br>CATGCCGTAAACGATGAGTGCTAGGTGTTGGAGGGTTTCCGCCCTTCAGTGCCGCAGCTAACGCATTAAGCACTCCGCCT<br>GGGGAGTACGACCGCAAGGTTGAAACTCAAAGGAATTGACGGGGACCCGCACAAGCG
Uncultured *Lactobacillus* sp. gene for 16S ribosomal RNA, partial sequence, clone: wb-13 (LC036256.1)	AGTCGAACGAGCTGCGCCTAATGATAGTTGATGCTTGCATTAACTTGACTTAAGTTAGC<br>AGCGAGTGGCGAACTGGTGAGTAACACGTGGATAACCTGCCCAGAAGAAGGGGATAACACCTGGAAACAGATGCTAATAC<br>CGTATAACAACGAAAACCACATGGTTTTCGTTTGAAAGATGGCCTTTGTGCTATCGCTTTTGGATGGATCCGCGGCGCAT<br>TAGCTAGTTGGTGAGATAAAGGCTCACCAAGGCAATGATGCGTAGCCGACCTGAGAGGGTAATCGGCCACATTGGGACTG<br>AGACACGGCCCAGACTCCTACGGGAGGCAGCAGTAGGGAATCTTCCACAATGGACGAAAGTCTGATGGAGCAATGCCGCG<br>TGAGTGAAGAAGGGTTTCGGCTCGTAAAACTCTGTTGTTAGAGAAGAACGGGCGTGAGAGTAACTGCTCACGTCGCGACG<br>GTATCTAACCAGAAAGTCACGGCTAACTACGTGCCAGCAGCCGCGGTAATACGTAGGTGGCAAGCGTTGTCCGGATTTAT<br>TGGGCGTAAAGCGAGCGCAGGCGGTCTTCTAAGTCTGATGTGAAAGCCTTCGGCTTAACCGGAGAAGTGCATCGGAAACT<br>GGGAAACTTGAGTGCAGAAGAGGACAGTGGAACTCCATGTGTAGCGGTGAAATGCGTAGATATATGGAGGAACACCAGTG<br>GCGAAGGCGGCTGTCTAGTCTGTAACTGACGCTGANGCTCGAAAGCATGGGTAGCAAACAGGATTAGATACCCTGGTAGT<br>CCATGCCGTAAACGATGAGTGCTAGGTGTTGGAGGGTTTCCGCCCTTCAGTGCCGCAGCTAACGCATTAAGCACTCCGCC<br>TGGGGAGTACGACCGCAAGGTTGAAACTCAAAGGAATTGACGGGGACCCGCACAAGCG

Table S2. Average relative abundances (in arbitrary units) of individual CHC species from conventional and germ-free females.

	Conventional females		Germ-free females	
**CHC species[1]**	**Mean (N = 3)**	**SD[2]**	**Mean (N = 3)**	**SD**
nC21	0.26	0.03	0.27	0.05
7,11-TD	0.66	0.06	0.49	0.08
9-T	0.34	0.03	0.34	0.03
7-T	1.31	0.15	1.33	0.01
5-T	0.09	0.02	0.08	0.02
nC23	8.03	1.43	8.86	1.40
2-MeC24	4.38	0.65	3.34	0.43
7,11-PD	0.39	0.15	0.33	0.03
9-P	5.70	0.43	5.77	0.23
7-P	3.12	0.09	3.29	0.09
5-P	0.34	0.06	0.38	0.01
nC25	9.00	2.50	9.75	1.59
2-MeC26	31.50	2.63	28.67	3.98
7,11-HD	15.85	1.41	15.30	1.38
9-H	5.05	0.57	5.28	0.50
7-H	4.86	0.61	6.04	0.10
2-MeC28	9.54	1.46	11.44	0.48
7,11-ND	10.20	1.06	12.16	1.25
nC29	0.28	0.08	0.48	0.04
2-MeC30	0.62	0.11	0.93	0.11

[1]Number indicates double bond position for alkenes and dienes and branching position for branched compounds; TD: tricosadiene; PD: pentacosadiene: HD: heptacosadiene; ND: nonacosadiene; T: tricosene; P: pentacosene; H: heptacosene; Me: methyl branched.
[2]SD: standard deviation
